# Tai Chi Movement Recognition and Precise Intervention for the Elderly Based on Inertial Measurement Units and Temporal Convolutional Neural Networks

**DOI:** 10.3390/s24134208

**Published:** 2024-06-28

**Authors:** Xiongfeng Li, Limin Zou, Haojie Li

**Affiliations:** 1 School of Physical Education and Sports, Beijing Normal University, Beijing 100875, China; 15513200878@xztu.edu.cn (X.L.); 202121070037@mail.bnu.edu.cn (H.L.); 2 Department of Physical Education, Xinzhou Normal University, Xinzhou 034000, China; 3 College of Physical Educantion, Jinggangshan University, Ji’an 343009, China

**Keywords:** tai chi, movement recognition, inertial measurement units, temporal convolutional neural networks, elderly intervention

## Abstract

(1) Background: The objective of this study was to recognize tai chi movements using inertial measurement units (IMUs) and temporal convolutional neural networks (TCNs) and to provide precise interventions for elderly people. (2) Methods: This study consisted of two parts: firstly, 70 skilled tai chi practitioners were used for movement recognition; secondly, 60 elderly males were used for an intervention study. IMU data were collected from skilled tai chi practitioners performing Bafa Wubu, and TCN models were constructed and trained to classify these movements. Elderly participants were divided into a precision intervention group and a standard intervention group, with the former receiving weekly real-time IMU feedback. Outcomes measured included balance, grip strength, quality of life, and depression. (3) Results: The TCN model demonstrated high accuracy in identifying tai chi movements, with percentages ranging from 82.6% to 94.4%. After eight weeks of intervention, both groups showed significant improvements in grip strength, quality of life, and depression. However, only the precision intervention group showed a significant increase in balance and higher post-intervention scores compared to the standard intervention group. (4) Conclusions: This study successfully employed IMU and TCN to identify Tai Chi movements and provide targeted feedback to older participants. Real-time IMU feedback can enhance health outcome indicators in elderly males.

## 1. Introduction

With the increasing trend of population aging worldwide, health issues of the elderly are a growing concern. As they age, older adults commonly face physical health challenges, which include vascular health problems [1], decreased musculoskeletal function [2], and weakened balance [3]. These physical problems not only affect the quality of life of older adults but also increase the risk of developing serious diseases such as cardiovascular disease [4]. Of particular concern is the fact that the body of older adults gradually loses vigor and elasticity as they age, leading to exercise as an important means of maintaining health [5]. However, older adults face challenges in choosing exercise programs and mastering exercise techniques and are prone to problems such as inaccurate exercise postures and inappropriate training methods, leading to poor exercise results and even injuries [6,7].

As a traditional Chinese fitness exercise, taijiquan has attracted more and more older people to participate in its unique gentle movements [8]. Studies have shown that taijiquan practitioners exhibit significant improvements in mental health [9], physiological function [10], and quality of life [11]. Despite the increasing popularity of taijiquan among older adults, relatively few studies have been conducted to quantitatively analyze the technique of its movements. Specifically, studies on the characteristics of taijiquan movements and their association with health in older adults are particularly scarce. This situation limits the development of individualized instruction and interventions for taijiquan practice. For example, Kammerlander [12] found that older adults may not achieve optimal health outcomes and may even be at increased risk of injury due to inaccurate postures or irregular movements [13]. Therefore, an in-depth technical analysis of taijiquan movements and their integration with the health status of older adults is of great practical importance. In order to solve this problem, the introduction of sensor technology, especially inertial measurement units (IMUs), has become an innovative approach. IMUs are able to accurately capture taijiquan movement data in real time, which provides the possibility of a detailed analysis of taijiquan movements. Roell [14] demonstrated that the use of IMUs can effectively recognize human movement movements and provide older adults with more accurate training Instruction.

The application of sensor technology in the field of sports is becoming increasingly popular, and the inertial measurement unit (IMU), as an important sensor, is able to capture human movement data in real time and accurately [15]. IMU has the advantages of being small, low cost, and easy to carry [16,17], and the application of IMU in the field of sports is also receiving increasing attention. For example, studies have shown that sports movements such as running involve multiple aspects, such as body posture, movement speed, and angle, while traditional manual assessment methods are subjective and uncertain. Using IMU technology, objective monitoring and quantitative analysis of running lower limb movements can be realized, which improves the accuracy of research and guidance [18]. In addition, research has shown that smart bracelets equipped with IMU sensors can monitor exercise movements in real time and provide real-time feedback to help practitioners correct their posture and improve the quality of their movements [19]. The application of IMU sensors in exercise training can also promote health management and disease prevention. By monitoring the status during exercise, potential health problems can be detected in time and targeted for intervention and guidance, thus improving the quality of life of the elderly [20]. By carrying IMU sensors for taijiquan movement recognition and feature analysis, accurate monitoring and quantitative assessment of taijiquan movements can be achieved, providing a scientific basis and personalized guidance for taijiquan training of the elderly.

Temporal convolutional neural network (TCN), as an emerging neural network structure, has shown excellent performance in the field of time series data analysis. It has the advantages of capturing long-term movement actions [21], efficient parallel computation and parameter sharing [22], and has great potential for application in the field of sports [23]. Combined with IMU data, the recognition and analysis of taijiquan movements using TCN can more accurately capture the characteristics of taijiquan movements and provide more precise intervention and guidance for taijiquan training for the elderly.

The novelty of the study is as follows: (1) For the first time, inertial measurement units (IMUs) and temporal convolutional neural networks (TCNs) were used to recognize taijiquan movements. This provides a new technical tool for tai chi movement recognition and intervention. (2) Combining neural network technology and taijiquan practice provides a scientific basis for health management and quality of life for the elderly. Through detailed identification and precise intervention of taijiquan movements, personalized guidance is provided to the elderly to increase their interest and engagement in taijiquan practice and promote physical and mental health. (3) Taijiquan movements are analyzed through IMU and TCN to more accurately capture the characteristics of taijiquan movements and provide more precise interventions and guidance for taijiquan training for older adults.

Previous studies on taijiquan movement recognition have mainly used traditional methods of biomechanical characterization of movement, such as infrared motion capture, high-speed video cameras, and surface electromyography, to capture the kinematic and kinetic characteristics of movements [24,25]. However, there are some problems with these methods. First, data acquisition is relatively cumbersome and requires testing in specific experimental environments, limiting the scale of data acquisition and the scope of application. Second, these methods are difficult to apply to large-scale groups and cannot meet the needs of practical applications. In contrast, our study employs an inertial measurement unit (IMU) for taijiquan movement recognition. The IMU has many advantages. First, the compact size, low cost, and portability of IMUs make them ideal for use in sports. Second, there is no precedent for using IMUs in the field of taijiquan, which is what makes our study innovative. By using IMUs for movement recognition, we can quickly and accurately obtain data on taijiquan movements without relying on traditional complex equipment and experimental environments. Our study demonstrates the superiority of the method of using IMUs for taijiquan movement recognition through highly accurate movement recognition. This method is not only fast and convenient but also has a high accuracy rate, which can provide precise guidance and feedback to practitioners of taijiquan. Therefore, our study is highly innovative in the field of traditional Chinese sports and exercise, and it is also an important innovation for the field of exercise.

Currently, there is no research on the IMU and TCN analysis of taijiquan movements among the elderly. This study aims to utilize this innovative technological tool for the first time to provide detailed identification and precise intervention of taijiquan movements in the elderly, providing scientific support and personalized guidance for the health of the elderly, which is of great theoretical and practical significance. In addition, this study will help to promote the popularization and promotion of the taijiquan movement among the elderly population. Scientific and accurate movement identification and personalized guidance can increase the interest and participation of the elderly in taijiquan and thus promote the physical and mental health of the elderly population. Therefore, this study has important theoretical and practical significance and has a profound impact on health management and the quality of life of the elderly.

## 2. Participants and Methods

### 2.1. Participants 

In the present study, two distinct participant groups were enrolled, each with specific inclusion and exclusion criteria. The initial 70 skilled tai chi practitioners, aged between 20 and 40 years, were selected to assess movement recognition. These individuals had accumulated more than two years of dedicated tai chi practice and demonstrated expert proficiency in executing the intricate Bafa Wubu sequence. Basic subject information (Table 1).

The secondary 60 elderly male participants, aged between 60 and 70 years, were selected based on their overall good health status and lack of mobility restrictions, thereby guaranteeing their fitness for engaging in the targeted exercise regimen. Eligibility criteria for this group included the capacity to provide informed consent and the absence of any significant health issues that might impact their participation. Individuals with a history of neurological disorders, severe cardiovascular disease, or other medical conditions that contraindicated safe involvement in the study’s physical activities were excluded.

All participants provided informed consent subsequent to receiving comprehensive information regarding the study’s aims, methodologies, potential risks, and benefits. The research protocol underwent a comprehensive review by the Institutional Ethics Committee at the Beijing Normal University, adhering strictly to the tenets of the Declaration of Helsinki. Following this review, the protocol was granted ethical clearance under approval number 20230221.

### 2.2. Method

#### 2.2.1. Study Design

**Figure 1:**
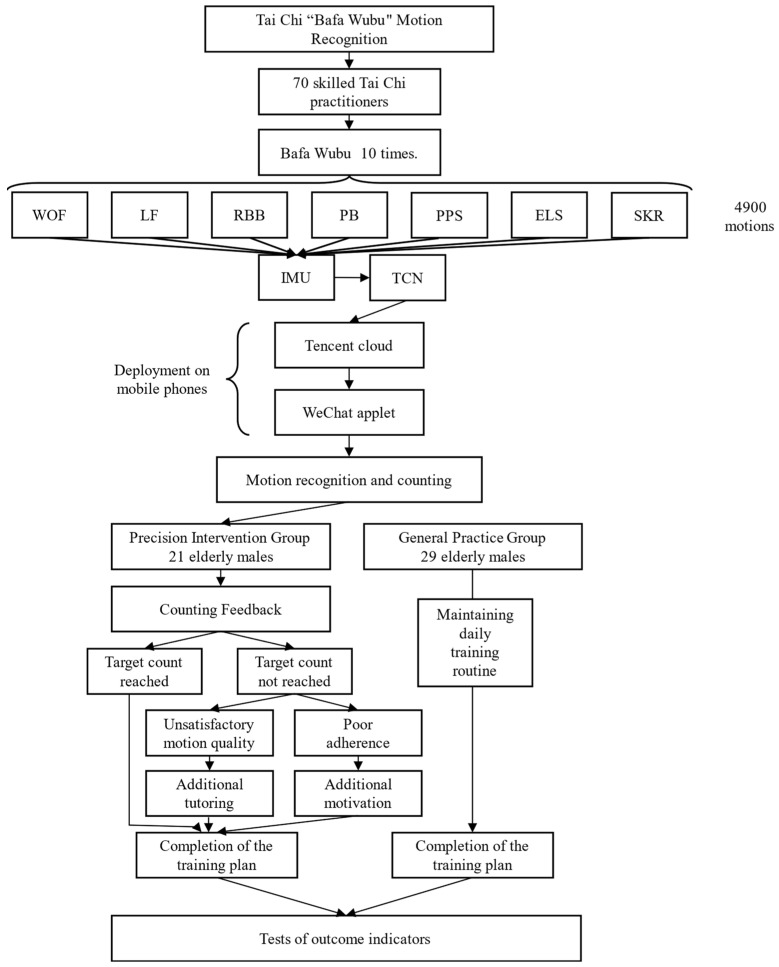
Experimental flow chart.

The study employed a two-phase design. In the cross-sectional phase, IMU data were collected from 70 adept tai chi practitioners as they executed the Bafa Wubu routine. Each participant performed the sequence ten times, thereby yielding a dataset of 70 individual movements per person. This part of the study aimed to establish a robust reference for tai chi movement recognition through the captured IMU data. In the subsequent interventional phase, the 60 elderly male participants were randomly assigned to either a precision intervention group or a standard intervention group. The precision intervention group received supplementary, tailored guidance or encouragement based on real-time feedback from their IMU-derived movement data on a weekly basis. Conversely, the standard intervention group maintained their usual daily routines without such personalized feedback. Both groups were evaluated for changes in balance, grip strength, quality of life, and depression indicators before and after the intervention period (Figure 1).

#### 2.2.2. Bafa Wubu Formulation in Tai Chi Exercise

**Figure 2:**
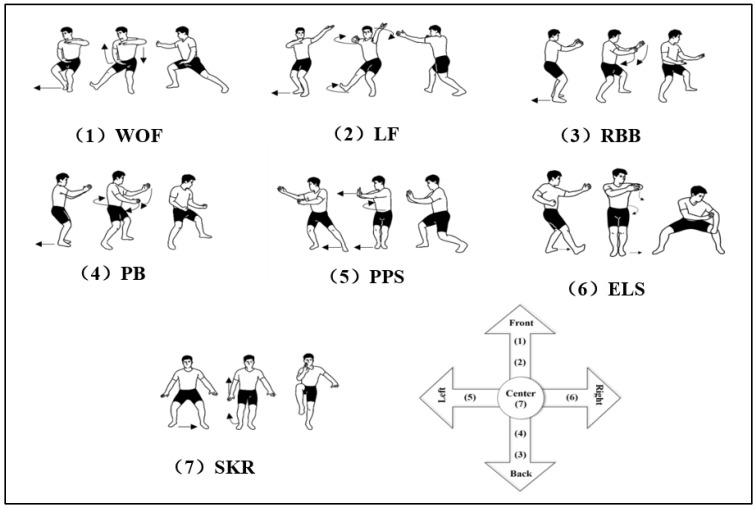
Schematic diagram of the motion of the Bafa Wubu. Note: Abbreviations: WOF: progressive ward-off; LF: progressive press; RBB: retreating roll-back; PB: retreating pluck; PPS: lateral push and pull; ELS: lateral elbow and lean; SKR: static knee lift. The black arrows in the figure show the trajectory of the direction of motion.

In the context of tai chi, the Bafa Wubu sequence represents a fundamental routine that encompasses a variety of footwork techniques. This traditional form integrates five distinct types of movement, collectively known as Jin (advancing), Tui (retreating), Gu (turning left), Pan (turning right), and Ding (steadying or neutral). As illustrated in Figure 2, the Bafa Wubu sequence is structured by harmonizing these fundamental steps with the eight key principles of tai chi, resulting in seven distinct motion sets: WOF (progressive ward-off), LF (progressive press), RBB (retreating roll-back), PB (retreating pluck), PPS (lateral push and pull), ELS (lateral elbow and lean), and SKR (static knee lift). Specifically, WOF and LF involve forward movement; RBB and PB involve backward movement; PPS and ELS involve lateral movement; and SKR represents a stable, immobile stance. These sets encapsulate the 13 fundamental techniques intrinsic to tai chi, each with unique characteristics regarding the direction of movement, methods of force generation, and the specific health benefits they provide. Consequently, these seven motion sets were deemed appropriate for the focus of this study.

#### 2.2.3. IMU Data Acquisition and Processing

This study employed the WitMotion Bluetooth 2.0 Six-Axis, Accelerometer–Gyroscope and Pitch–Roll–Yaw Angle Sensor (Wit Intelligence, Shenzhen, China), which was affixed to the left upper arm of the participants. Accelerometers have a range of ±16 g and gyroscopes ±2000°/s. The IMU was affixed to the upper left arm, and two IMUs were utilized in rotation to ensure the continuity of data collection. During the execution of each movement in the Bafa Wubu routine, raw inertial measurement unit (IMU) data were meticulously captured, providing a comprehensive record of motion dynamics. 

The raw IMU signals were subjected to a low-pass filtering process in order to eliminate high-frequency noise. A cutoff frequency of 5 Hz was implemented in order to retain relevant movement information while discarding unwanted noise components. Following the low-pass filtering of the raw IMU signals, the filtered data were subsequently down-sampled to 50 Hz. This down-sampling rate was selected to align with the sampling capabilities of some smartphones, which typically have a 50 Hz sampling frequency. By adjusting the data to this rate, the resulting model is better suited for deployment on mobile platforms, thus expanding the potential applications and accessibility of the technology.

Subsequently, the filtered data were segmented using a 2 s window to isolate individual movements. In order to ensure sufficient context for movement recognition and to account for motion transitions, a 50% overlap rate was adopted in the segmentation procedure. This approach facilitated the extraction of meaningful and temporally coherent features from the continuous data stream for further analysis.

#### 2.2.4. Neural Network Construction

In this research, a temporal convolutional neural network (TCN) model was meticulously constructed using TensorFlow to process data from a six-axis inertial measurement unit (IMU) sensor, with the aim of classifying tai chi movements. The TCN architecture was designed with multiple homogeneous blocks, each comprising a 1D convolutional layer to capture temporal relationships, a batch normalization layer to enhance training stability and efficiency, and a dropout layer for regularisation. The Rectified Linear Unit (ReLU) activation function was used to introduce non-linearity into the model.

This model was chosen for its effectiveness in extracting temporal features from IMU sensor data, as the TCN is adept at capturing the causal relationships within time series that are crucial for accurate motion recognition. The TCN model was designed with no residual connections, maintaining simplicity while ensuring sufficient capacity to learn the temporal dynamics of tai chi movements. The input to the TCN is IMU data formatted as a 3D array in the structure of samples, time, and channels.

The model culminated in a fully connected layer that integrated the learned features to classify tai chi movements. Hyperparameter optimization, including the number of blocks and neurons in the fully connected layer, the dropout rate, and the learning rate, was performed using an Optuna method to fine-tune the model’s performance. The model finally employed 20 blocks, along with a dropout rate of 0.44. A total of 1000 training epochs were run to ensure convergence of the loss function, which is critical for robust training of the neural network. The dataset was meticulously partitioned into a two-thirds portion for training and a one-third portion for validation.

**Figure 3:**
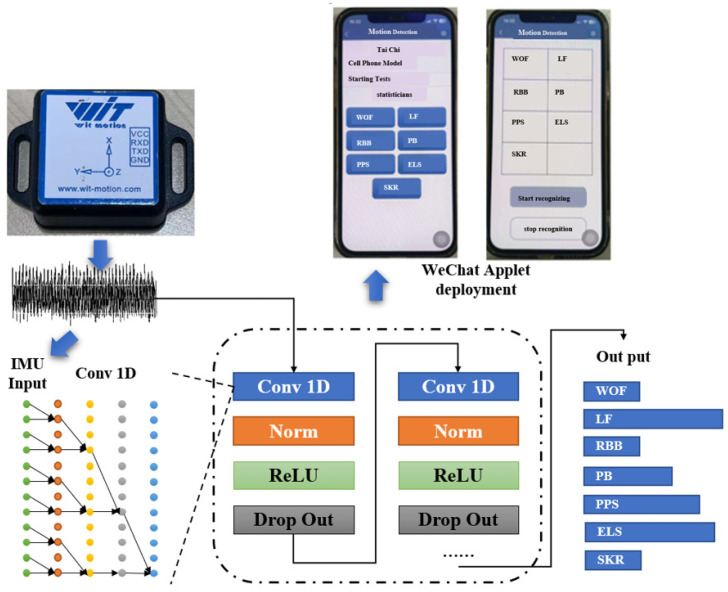
Structural diagram of a temporal convolutional neural network.

The performance of the TCN model was quantitatively assessed using a confusion matrix, which was employed to evaluate the classification accuracy of each tai chi movement. The accuracy metric was employed to measure the model’s overall performance in distinguishing between different movements. This provided a comprehensive assessment of its predictive capabilities in the context of tai chi motion classification based on IMU data (Figure 3).

### 2.3. Deployment on Mobile Devices

#### 2.3.1. WeChat Applet Deployment

The user interface within the WeChat applet, developed by Tencent Holdings Ltd. (Shenzhen, China), allows users to select their operating system, differentiating between iOS and Android users. Once the motion detection process is initiated, the applet is designed to send real-time motion detection data to the server at one-second intervals.

#### 2.3.2. Server-Side Deployment

Deployed on Tencent Cloud, a service provided by Tencent Holdings Ltd. (Shenzhen, China), the server is equipped with a Python-based Flask framework to handle data reception and transmission. This framework also facilitates the invocation of the temporal convolutional neural network (TCN) model for motion detection and real-time computation. Once a movement has been identified, an incrementing function is used to count the number of movements detected.

Participants initiate the mobile application by entering the required information, and the WeChat applet proceeds to recognize and count the tai chi movements in real time. Finally, the participant’s movement counts and other relevant data are stored on Tencent’s cloud server for subsequent analysis and record keeping.

#### 2.3.3. Exercise Intervention Program

A group of 60 elderly males was stratified into two distinct groups: the precision intervention group and the standard intervention group. Participants in the precision intervention group were provided with a mobile phone carrier bag designed to be worn on the left upper arm to facilitate inertial measurement unit (IMU) data collection.

Both groups underwent an 8-week tai chi intervention program consisting of three sessions per week. Each session was structured to include a 5 min warm-up, followed by 30 min of continuous practice, and concluded with a 5 min cool-down.

For the precision intervention group, the end of the 30 min exercise was marked by a dedicated 4 min IMU data collection period, coinciding with the performance of a full set of tai chi movements. Using a smartphone, the intervention included real-time analysis and counting of movements. The participants received weekly feedback on the motion recognition and counts. Any discrepancies between the recognized counts and the designed distribution, which indicated that each movement was expected to occur an average of seven times, indicated either a lack of mastery of the movement technique or poor compliance. Those who demonstrated a lack of technique mastery were provided with additional coaching during the subsequent training session, while those with poor compliance were encouraged to improve their adherence.

At the end of the program, the precision intervention group had a dropout rate of 9 participants, resulting in 21 individuals completing the intervention. Conversely, the standard intervention group had a dropout rate of 1 participant, with a total of 29 individuals completing the intervention. The statistical test power of this sample was 80 percent after a post hoc test by Gpower 3.1.

#### 2.3.4. Health Outcome Measures

In this study, a suite of health outcome measures were employed to assess various aspects of the elderly participants’ health, including balance, grip strength, quality of life, and depression. Balance was evaluated using the one-leg stand test with eyes closed; this is a standard method for evaluating postural stability. Each participant was equipped with a balance meter (Model HK6000, Hengkang Jiaye, Shenzhen, China), which provided precise and standardized measurements of their balance capabilities.

Grip strength, a reliable indicator of upper limb strength and overall muscular health, was assessed using a hand dynamometer (HK6800, Hengkang Jiaye, Shenzhen, China). Participants were instructed to perform a single-grip test with their dominant hand, and the mean value from three consecutive trials was recorded to account for test-retest reliability.

Quality of life was evaluated using the 12-Item Short Form Survey (SF-12), a concise and widely used instrument that captures key dimensions of physical and mental health, offering a quick yet comprehensive snapshot of an individual’s health-related quality of life. The alpha reliability of SF-12 in this study was tested to be 0.84.

Psychological depression was screened using the Beck Depression Inventory (BDI), a psychometrically validated tool for measuring the presence and severity of depressive symptoms. The alpha reliability of BDI in this study was tested to be 0.89.

### 2.4. Statistical Analysis

Descriptive statistics were initially conducted to summarize the data, followed by a normality test to ascertain the distributional properties of the outcome variables. The normality of the data was examined using the Shapiro–Wilk test. A mixed-effects analysis of variance (ANOVA) was employed to explore the effects of the between-subjects factor (group allocation: precision intervention group vs. standard intervention group) and the within-subjects factor (time: pre- and post-intervention) on the outcome variables. The statistical analyses were performed using IBM SPSS26.0 Statistics software. The conventional threshold of 0.05 was employed to determine the statistical significance of the results.

## 3. Results

**Figure 4:**
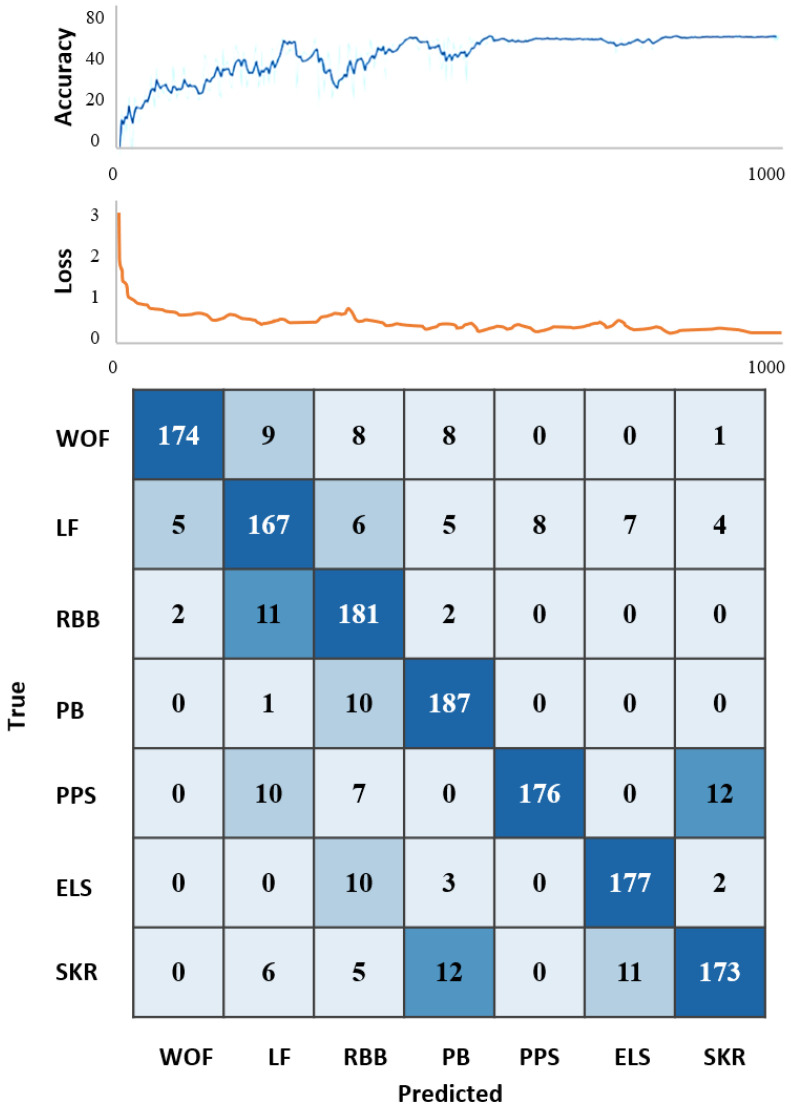
Training loss curves for motion recognition and the confusion matrix. Accuracy represents the accuracy change curve at 1000 epochs of training, and loss is the value of the loss function at 1000 epochs of training. Note: Abbreviations: WOF: progressive ward-off; LF: progressive press; RBB: retreating roll-back; PB: retreating pluck; PPS: lateral push and pull; ELS: lateral elbow and lean; SKR: static knee lift. The numeric values represent the number of specific categories, the higher the value, the darker the colour.

Table 2 and Figure 4 demonstrate that the TCN is capable of accurately recognizing WOF motion with an accuracy of 87.0%, LF motion with an accuracy of 82.6%, RBB motion with an accuracy of 92.3%, PB motion with an accuracy of 94.4%, PPS motion with an accuracy of 85.8%, ELS motion with an accuracy of 92.1%, and SKR motion with an accuracy of 83.6%.

The results presented in Table 3 demonstrate that both the precision intervention group and standard intervention group demonstrate a significant increase in grip strength, SF-12 scores, and a significant decrease in BDI following the 8-week intervention. With regard to balance indicators, only the precision intervention group exhibited a significant increase, and the precision intervention group exhibited a significantly higher score than the standard intervention group following the intervention.

## 4. Discussion

We found that for different taijiquan moves, the TCN (Temporal Convolutional Network) showed different levels of accuracy in recognizing them. Most notably, the TCN achieved 94.4% accuracy in recognizing the PB action, which indicates that the network is highly reliable in recognizing this particular taijiquan action. This was followed by the RBB action and the ELS action, which achieved 92.3% and 92.1% accuracy, respectively, also showing the excellent performance of the TCN in recognizing these two actions. However, the recognition accuracy of LF actions and SKR actions is lower, 82.6% and 83.6%, respectively, which may be due to the fact that these actions have more complex motion features, making the neural network face a greater challenge in recognizing them. In addition, the recognition accuracies of WOF and PPS actions were 87.0% and 85.8%, respectively, which were in the middle of the range, showing the relatively average performance of TCNs in the recognition of these actions. Our study identified differences in the recognition accuracy of different movements in Taijiquan, which may be related to the complexity of the movements and the movement characteristics. This is in line with Huang’s study, which concluded that the complexity of actions and motion features have a significant effect on the recognition ability of neural networks. Their study observed the difference between the recognition accuracy of different sports actions in soccer and pointed out that the complexity of the action may be an important factor for this difference [26]. In addition, this may be related to the fact that complex tai chi movements tend to involve more joint movements and body coordination, which increases the difficulty of neural networks in capturing and understanding movement features [27]. In contrast, simple movements may have clearer and more recognizable movement patterns, which improves the recognition accuracy of neural networks [28]. This finding emphasizes the importance of considering movement complexity in the design of taijiquan movement recognition systems. Future research can further explore the differences in motion characteristics between different movements and improve the recognition ability for complex movements by optimizing the neural network structure and training algorithms. Overall, although there is still room for improvement, the recognition accuracy of taijiquan in this study, which employed a single IMU and the TCN algorithm, has reached a satisfactory level. This outcome demonstrates that the TCN algorithm has considerable potential for the analysis of IMU data.

Another result of our study found that balance improvement was more prominent through precision taijiquan intervention relative to standard taijiquan intervention. Specifically, there was a significant improvement in the performance of the precision intervention group in the balance test, where previous studies have shown that taijiquan practice is widely recognized to enhance balance, especially among older adults [29]. This present study found that this effect was further enhanced by a precision intervention through neural networks for the recognition of taijiquan movements in older adults. The precise taijiquan intervention may illustrate the necessity and reliability of taijiquan technical movement recognition with neural networks through finer movement control and higher concentration.

In addition, in this study, both the precision tai chi intervention group and the standard tai chi intervention group showed significant improvements in grip strength. Grip strength is one of the most important indicators for assessing hand and overall body strength. Its improvement may reflect the enhancement of muscle strength and body core stability by tai chi practice. The increase in grip strength is important for activities that require hand strength in daily life (e.g., lifting objects, walking, etc.) [30]. This result is consistent with previous studies. For example, Alqahtani’s [31] study found that tai chi exercise significantly improved upper limb strength in older adults. This further emphasizes the potential of tai chi in promoting health in older adults. In addition, a study by Kato [32] showed that grip strength is closely related to life function and independence in older adults. Therefore, improvements in grip strength may reduce the risk of reduced quality of life due to limitations in daily activities. Thus, the results of this study further emphasize the important role of tai chi in the elderly population, especially in promoting muscle strength and maintaining daily living functions. This provides an important scientific basis for the practical application of taijiquan interventions and suggests the potential value of taijiquan in the health management of older adults.

In this paper, we investigate the innovative finding that precision taijiquan intervention through neural network recognition of taijiquan movements was found to be significantly better than conventional taijiquan intervention in terms of quality of life and depression level. This evidence suggests that precision tai chi intervention not only enhances physical functioning but also positively affects quality of life and psychological well-being in older adults. The precision tai chi intervention achieved significant results in improving quality of life and depression levels compared to the conventional tai chi intervention. This finding is consistent with the findings of Li [33], who found that tai chi practice had significant improvements in the quality of life and mental health of older adults. This study found that the precision intervention group performed more prominently in these areas, which further confirms the effectiveness of the precision intervention. Improving quality of life and depression levels is important for older adults. As they age, older adults may face problems such as decreased physical function, reduced social activities, and increased psychological stress [34]. Therefore, precise intervention through tai chi practice can bring multiple benefits. In addition, reducing depression is one of the important benefits of precision tai chi intervention. Tai chi practice focuses on breathing regulation and meditation, which can help older adults reduce negative emotions such as anxiety and depression and improve mental health [35,36]. Therefore, the results of this study provide a solid scientific basis for the application of precision taijiquan intervention in the elderly population. Through precise intervention of taijiquan movements, we can not only improve the physical health of the elderly but also enhance their quality of life and mental health, providing an effective intervention for the overall health of the elderly.

This study demonstrates important advances in taijiquan movement recognition and precision intervention. It not only technically improves the level of taijiquan movement recognition and precise intervention but, more importantly, provides a solid scientific basis for the practical application of taijiquan in the elderly population. By combining neural network technology and taijiquan practice, this study makes an important contribution to the promotion of health management and quality of life for the elderly and demonstrates the broad prospects and potential of taijiquan in the field of elderly health.

### 4.1. Advantage

(1) This study is the first to use IMU and TCN to analyze taijiquan movements, providing a new technological tool for taijiquan movement recognition and intervention among older adults.

(2) By combining neural network technology and taijiquan practice, the study provides a scientific basis for health management and quality of life in the elderly population.

(3) By identifying in detail and intervening precisely in taijiquan movements, the study provides personalized instruction for older adults to help them better participate in taijiquan practice, increase interest and engagement, and promote physical and mental health.

### 4.2. Limitations

Despite the innovative and scientifically significant nature of this study in terms of tai chi movement identification and intervention, there is a need to further expand the sample size, increase external validity, and improve the data collection methodology to better support the use of tai chi in older adult populations. Therefore, in order to better support the use of tai chi in the elderly population, there is a need to expand the sample size, increase external validity, and improve data collection methods. These improvements could increase the reliability and applicability of the study to better support the use of tai chi in the health management of older adults.

## 5. Conclusions

This study provides an important exploration and innovation on the recognition and intervention of taijiquan movements by combining neural network technology and taijiquan. By analyzing taijiquan movements through IMU and TCN, accurate recognition and precise intervention of taijiquan movements can be achieved. The results of the study show that TCN exhibits high accuracy in recognizing taijiquan movements. In addition, the precise intervention of tai chi movements showed significant improvement in balance in older adults. It also demonstrated the potential and importance of taijiquan in promoting the physical and mental health of older adults. Therefore, this study has important theoretical and practical significance and has a profound impact on the health management and quality of life improvement of older adults.

## Figures and Tables

**Table 1:** Basic characteristics of participants.

	Precision Intervention Group (n = 21)	Standard Intervention Group (n = 29)	Skilled Tai Chi Practitioner (n = 70)	*p*-Value for Differences between Groups	*p*-Value for Normality Test
Age	65.2 ± 4.2	64.9 ± 3.7	36.1 ± 3.5	N/A	N/A
Height (cm)	162.85 ± 6.53	164.43 ± 7.82	169.64 ± 11.85	N/A	N/A
Weight (kg)	69.31 ± 6.92	70.87 ± 7.88	75.48 ± 7.11	N/A	N/A
Blance (s)	8.61 ± 4.21	7.06 ± 3.13	N/A	0.14	0.33/0.07
Gripstrength (kg)	36.17 ± 4.82	33.69 ± 6.88	N/A	0.16	0.97/0.75
SF-12	30.39 ± 1.61	30.07 ± 4.68	N/A	0.76	0.42/0.20
BDI	19.13 ± 8.03	19.92 ± 7.07	N/A	0.71	0.53/0.98

Note: N/A indicates not applicable; *p*-values for normality tests for the two groups are indicated by numbers/numbers.

**Table 2:** Accuracy of motion recognition.

Motion	True Count	False Count	Accuracy
WOF	174	26	87.0%
LF	167	35	82.6%
RBB	181	15	92.3%
PB	187	11	94.4%
PPS	176	29	85.8%
ELS	177	15	92.1%
SKR	173	34	83.6%

Note: Abbreviations: WOF: progressive ward-off; LF: progressive press; RBB: retreating roll-back; PB: retreating pluck; PPS: lateral push and pull; ELS: lateral elbow and lean; SKR: static knee lift.

**Table 3:** Indicators of health outcomes before and after the intervention.

	Precision Intervention Group (n = 21)			Standard Intervention Group (n = 29)			Interaction	
	Pre	Post	P	Pre	Post	P	P	η^2^p
Blance (s)	8.61 ± 4.21	10.72 ± 3.50	0.010	7.06 ± 3.13	7.21 ± 2.18 *	0.136	0.059	0.075
Gripstrength (kg)	36.17 ± 4.82	39.78 ± 3.22	0.000	33.69 ± 6.88	38.98 ± 5.15	0.000	0.002	0.196
SF-12	30.39 ± 1.61	31.46 ± 2.57	0.005	30.07 ± 4.68	30.87 ± 5.94	0.005	0.670	0.004
BDI	19.13 ± 8.03	16.06 ± 3.44	0.000	19.92 ± 7.07	17.90 ± 5.90	0.001	0.241	0.030

Note: Abbreviations: SF-12, 12-Item Short Form Survey; BDI: Beck Depression Inventory; * indicates a significant difference compared to the precision intervention group.

## Data Availability

The data that support the findings of this study are available on request from the corresponding author.
